# Effects of Protonation, Hydroxylamination, and Hydrazination of g-C_3_N_4_ on the Performance of Matrimid^®^/g-C_3_N_4_ Membranes

**DOI:** 10.3390/nano8121010

**Published:** 2018-12-05

**Authors:** María Soto-Herranz, Mercedes Sánchez-Báscones, Antonio Hérnandez-Giménez, José I. Calvo-Díez, Jesús Martín-Gil, Pablo Martín-Ramos

**Affiliations:** 1 Department of Agroforestry Sciences, ETSIIAA, University of Valladolid, Avenida de Madrid 44, 34004 Palencia, Spain; msanchez@agro.uva.es; 2 SMAP, UA-UVa_CSIC, Associated Research Unit to CSIC, Universidad de Valladolid, Paseo Belén 7, 47011 Valladolid, Spain; tonhg@termo.uva.es (A.H.-G.); jicalvo@termo.uva.es (J.I.C.-D.); 3 Agriculture and Forestry Engineering Department, ETSIIAA, Universidad de Valladolid, Avenida de Madrid 44, 34004 Palencia, Spain; mgil@iaf.uva.es; 4 Department of Agricultural and Environmental Sciences, Instituto Universitario de Investigación en Ciencias Ambientales (IUCA), EPS, Universidad de Zaragoza, Carretera de Cuarte s/n, 22071 Huesca, Spain

**Keywords:** carbon nitride, CO_2_/CH_4_, gas separation, Matrimid^®^ 5218, mixed matrix membrane, O_2_/N_2_

## Abstract

One of the challenges to continue improving polymeric membranes properties involves the development of novel chemically modified fillers, such as nitrogen-rich 2-D nanomaterials. Graphitic carbon nitride (g-C_3_N_4_) has attracted significant interest as a new class of these fillers. Protonation is known to afford it desirable functionalities to form unique architectures for various applications. In the work presented herein, doping of Matrimid^®^ with protonated g-C_3_N_4_ to yield Matrimid^®^/g-C_3_N_4_ mixed matrix membranes was found to improve gas separation by enhancing the selectivity for CO_2_/CH_4_ by up to 36.9% at 0.5 wt % filler doping. With a view to further enhancing the contribution of g-C_3_N_4_ to the performance of the composite membrane, oxygen plasma and hydrazine monohydrate treatments were also assayed as alternatives to protonation. Hydroxylamination by oxygen plasma treatment increased the selectivity for CO_2_/CH_4_ by up to 52.2% (at 2 wt % doping) and that for O_2_/N_2_ by up to 26.3% (at 0.5 wt % doping). Hydrazination led to lower enhancements in CO_2_/CH_4_ separation, by up to 11.4%. This study suggests that chemically-modified g-C_3_N_4_ may hold promise as an additive for modifying the surface of Matrimid^®^ and other membranes.

## 1. Introduction

CO_2_ is significantly present in mixtures where CH_4_ is the major and valuable component. Its removal from CH_4_ mixtures is particularly important in processes such as biogas upgrading or natural gas sweetening. Conventional industrial methods used for such CO_2_ removal include adsorption [1], and water scrubbing and absorption [2] processes. Nonetheless, separation processes mediated by membranes can generally offer advantages over aforementioned techniques in terms of low capital cost, ease of processing, small footprint area, high energy efficiency, and ease of preparation and control [3,4].

Another closely related burgeoning field is that of oxygen production from air separation, since it can be used in many environmental applications, such as in the combustion enhancement of natural gas or in coal gasification [5]. Although cryogenic distillation and pressure swing adsorption are the mainstream technologies for oxygen-enriched air production, the use of membranes has been deemed as specially promising.

Basu et al. [6] and Zhang et al. [7] published two reviews on membrane materials used for CO_2_/CH_4_ separation. Among them, and apart from zeolite SAPO-34-based membranes [8,9,10,11,12], polyimides (PI) were claimed to exhibit the best combination of permeability and selectivity, thermal and chemical stability, and water resistance in biogas upgrading. In a similar fashion, glassy polyimides have also proven to exhibit excellent O_2_/N_2_ selectivity [13].

The most popular commercially available PI, 3,3’-4,4’-benzophenone tetracarboxylic-dianhydride diaminophenylindane, better known as Matrimid^®^ 5218A, has been shown to combine a remarkable gas separation performance with other desirable features in terms of solubility, processability, thermal stability, chemical resistance, adhesion, film durability, and toughness. The state-of-the art based on this thermoplastic PI for gas separation applications has been recently covered in a thorough review by Castro-Muñoz et al. [14]. In terms of future trends to further enhance Matrimid’s properties, they insisted on the need to continue developing novel chemically modified fillers as a key challenge.

Amongst two-dimensional nanomaterials that are being explored for the development of high-performance separation membranes, discussed in a recent review by Zhu et al. [15], graphitic carbon nitride (g-C_3_N_4_) has attracted significant interest as a new class of filler. This mild bandgap metal-free organic semiconductor has remarkable applications as a catalyst for environmental pollution remediation [16,17].

Theoretical studies have preconized its suitability for H_2_ or He purification from other gases [18,19,20,21], and several experimental works have shown that it can be successfully used in the fabrication of hybrid membranes with enhanced properties. For instance, Hou et al. [22] reported a zeolitic imidazolate framework-8 (ZIF-8) hybrid membrane doped with g-C_3_N_4_ nanosheets for H_2_/CO_2_ separation, with an excellent selectivity. g-C_3_N_4_ nanosheets were also incorporated into the matrix of polymers of intrinsic microporosity (PIM-1) by Tian et al. [23], resulting in an increase in the selectivities for H_2_/CH_4_ and H_2_/N_2_.

As regards other membrane separation (non-gas) applications, Wang et al. [24] co-doped a polyamide (PA) forward osmosis membrane, based on a porous polyethersulfone substrate, with reduced graphene oxide and graphitic g-C_3_N_4_. By doing so, they obtained an osmotic water flux nearly 20% higher than that of the control membrane. Also in a PA nanofiltration membrane, modification with g-C_3_N_4_ nanosheets was shown to improve the permeability, antifouling properties, hydrophilicity, negative charge density, roughness and thermal stability [25]. Similar conclusions were reached for reverse osmosis PA membranes doped with acidified g-C_3_N_4_ by Gao et al. [26]. Doping g-C_3_N_4_ into a sodium alginate matrix or into poly(vinyl alcohol) also improved the performance of hybrid membranes for water/ethanol separation [27,28]; and doping of g-C_3_N_4_ co-impregnated with Cu^+^ and Fe^2+^ ions into a Pebax^®^ membrane improved gasoline desulfurization [29].

Nonetheless, to the best of the authors’ knowledge, there have been no reports on the utilization of g-C_3_N_4_ as a filler for Matrimid^®^ mixed matrix membranes (MMMs). The work presented herein addresses this literature gap by assessing the effects of protonation of g-C_3_N_4_ as well as its treatment with hydrazine monohydrate and with oxygen plasma on the performance of MMMs for CO_2_/CH_4_ and O_2_/N_2_ separation.

## 2. Materials and Methods

### 2.1. Materials

Matrimid^®^ 5218 polymer (CAS no. 104983-64-4) was obtained from Huntsman Chemical, Advanced Materials Americas Inc. (Los Angeles, CA, USA). Melamine cyanurate (CAS no. 37640-57-6) was supplied by Ferro-plast S.r.l. (Vimodrone, Italy) with a purity superior to 99%. *N*-*N*-Dimethylacetamide (DMAc; CAS no. CAS: 127-19-5; ≥99%) and hydrazine monohydrate (CAS no. 7803-57-8; 98%) were purchased from Sigma-Aldrich (Munich, Germany). Reagents were used as received, without any further purification.

### 2.2. Methods

#### 2.2.1. Synthesis of Graphitic Carbon Nitride (g-C_3_N_4_)

Graphitic carbon nitride was prepared by a modification of the thermal oxidation method [30], using melamine cyanurate as a starting material for the polymerization/condensation reaction [31]. The reagent was placed in a Vycor^®^ glass vial and heated at 650 °C for 4 h under aeration, with a cooling/heating ramp of 10 °C·min^−1^. The resulting yellow powder was transferred to an agate mortar and ground. Subsequently, the samples were exposed to ultrasonic treatment for approximately 2 h using a Branson (FisherScientific, Hampton, NH, USA) Sonifier 450. Samples were dried in an oven at 105 °C for 24 h to remove moisture.

#### 2.2.2. Synthesis of Protonated g-C_3_N_4_

**Figure 1:**
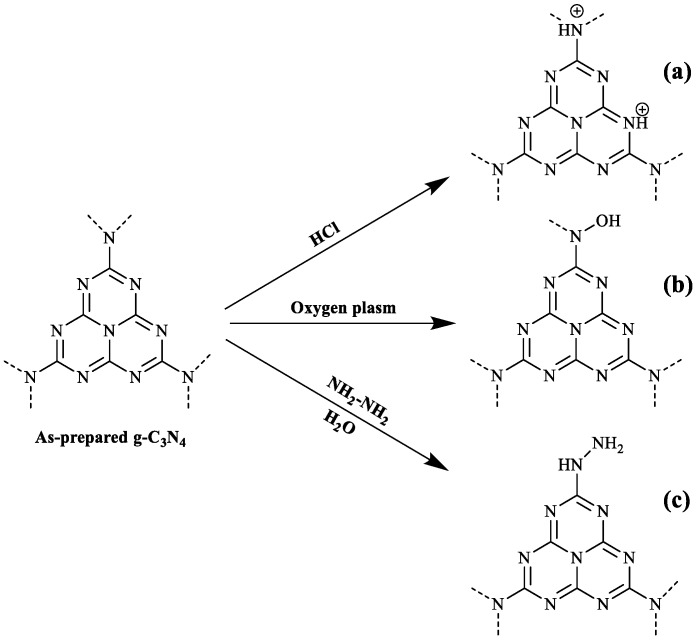
Scheme of the synthesis of: (**a**) protonated g-C_3_N_4_; (**b**) oxygen plasm treatment used to introduce hydroxylamine structure (N-OH) on the surface of g-C_3_N_4_ (hydroxylaminated g-C_3_N_4_); and (**c**) hydrazinated g-C_3_N_4_.

Due to the presence of abundant –C–N– bonds in the g-C_3_N_4_ framework, it can be easily protonated by HCl, resulting in a modification of the surface charge, from a negatively to positively charged (Figure 1a) [32]. The process consisted in adding 2 g of pristine g-C_3_N_4_ sample to a 12 mL diluted 3% solution of HCl and treating it with ultrasound for 2 h. Samples were not subsequently washed. They were placed in an oven at 105 °C for approximately 24 h and were then ground.

#### 2.2.3. Modifications of g-C_3_N_4_ with Oxygen Plasma and Hydrazine Monohydrate

g-C_3_N_4_ is a laminar material with non-oxidized aromatic regions and aliphatic regions containing phenolic, carboxyl, and oxygen epoxide groups that make it hydrophilic in aqueous media. This behavior can be modified through treatment with oxygen plasma or with hydrazine monohydrate.

In the former approach, interfacial forces are improved via plasma treatment, an ecologically benign process that does not produce liquid chemical residues. When the plasma is used with oxygen gas, it dissociates in order to generate oxygen-containing radicals [33], which incorporate –OH, as hydroxylamine groups, into the surface of g-C_3_N_4_, improving its dispersibility [34] (Figure 1b). The oxygen plasma treatment was conducted using a Harrick Plasma (Ithaca, NY, USA) PDC-002 apparatus. 150 mg of g-C_3_N_4_ were placed on a Petri dish on a quartz sample holder within the plasma cavity. The operating power remained at 10.2 W, the chamber pressure was 300 Torr with an O_2_ flow of 35 cm^3^·min^−1^, and the treatment time was 90 min.

In the later approach, diazanyl group modified g-C_3_N_4_-NHNH_2_ (Figure 1c) was obtained according to the procedure described by Chen et al. [35]: 1 g of as-synthesized g-C_3_N_4_ was mixed with 20 mL water and 4 mL hydrazine hydrate, followed by stirring at 80 °C for 40 min.

#### 2.2.4. Preparation of the Matrimid/g-C_3_N_4_ Mixed Matrix Membranes

The Matrimid/g-C_3_N_4_ MMMs were prepared from a Matrimid commercial polymer solution doped with 0.5 wt % or 2 wt % g-C_3_N_4_ loading in 5 mL of DMAc solvent at adequate concentration (6% w/v) to achieve a reasonable viscosity. Working at such g-C_3_N_4_ loadings, previous work showed that measurable effects were obtained as compared with pristine Matrimid membranes, while much higher loadings led to an inhomogeneous distribution of g-C_3_N_4_ across the membrane film, thus compromising the mechanical stability of the resulting membranes. After stirring until complete homogenization, all solutions were sonicated for 5 min at an amplitude of 60% in a sonicator with a work/rest period of 20 s and 10 s. Next, membranes were prepared by the casting method following the protocol described by Recio et al. [36], using a glass plate at 25 °C. The resulting films were dried at 60 °C for 24 h to complete the removal of the solvent and were then placed in a vacuum oven, where they remained at 120 °C for 4 hours before raising the temperature to 180 °C for another 4–6 h. Finally, the membranes were separated from the glass plates and their thickness was determined using a Fischer (Sindelfingen, Germany) Dualscope MP0R. All thicknesses were in the 40–60 μm range. Three samples of each formulation were prepared, and at least two pieces of each membrane were tested. Their results were averaged, with a sample to sample variability in the usual range for laboratory prepared membranes (5–10%).

#### 2.2.5. Characterization Methods

Vibrational information of the dopant and the Matrimid/g-C_3_N_4_ MMMs was retrieved by using a Thermo Scientific (Waltham, MA, USA) Nicolet iS50 Fourier-Transform Infrared (FTIR) spectrometer. 8–10 mm Ø samples were cut from the films and their transmittance was measured at room temperature in a scanning range between 410 and 4000 cm^−1^, with a 1 cm^−1^ spectral resolution and 64 scans.

The morphology of the as-prepared samples was examined using field emission scanning electron microscopy (FESEM), with a FEI (Hillsboro, OR, USA) QUANTA 200F device.

Thermogravimetric analysis (TG) was used to determine the thermal and/or oxidative stability of the materials. TG experiments were performed with a Mettler Toledo (Columbus, OH, USA) DMA/SDTA 861 device, heating the samples from 50 °C to 850 °C at a heating rate of 10 °C·min^−1^, under a N_2_ flow of 20 cm^3^·min^−1^.

#### 2.2.6. Gas Separation Performance Measurement

The pure gas permeation tests were performed on an isochoric (constant volume, variable pressure) permeation system to measure the permeability properties of the membranes. Five pure gas species (He, N_2_, O_2_, CH_4_, and CO_2_) were applied as test gases, and the permeability and perm-selectivity of Matrimid/g-C_3_N_4_ MMMs were evaluated. Experiments were carried out at 35 °C using a constant feed pressure of 3 bar. To assure constant flow of each gas tested, desired pressure was maintained during 1 h and then flow measurements were taken by using a gas flow meter.

The permeability of a given membrane was calculated from Equation (1)

(1) $$P=\frac{Q\times L}{\Delta P\times A}$$

where *P* is the permeability in barrer (1 barrer = (10^−10^ cm^3^ (STP) × cm)/(cm^2^ × s × cmHg)), *Q* is the volumetric flow rate [cm^3^ (STP)/s], *L* is the membrane thickness [cm], Δ*P* is the pressure difference between two sides of the membrane [cmHg], and *A* is the effective membrane area [cm^2^]. The perm-selectivity *α*_A/B_ is defined as the ratio of the permeability coefficients of two gases *A* and *B* (*P*_A_ and *P*_B_), according to Equation (2)

(2) $$\alpha_{A/B}=\frac{P_{A}}{P_{B}}$$

## 3. Results and Discussion

### 3.1. Vibrational Characterization

#### 3.1.1. ATR-FTIR Spectra of the Modified g-C_3_N_4_ Fillers

**Figure 2:**
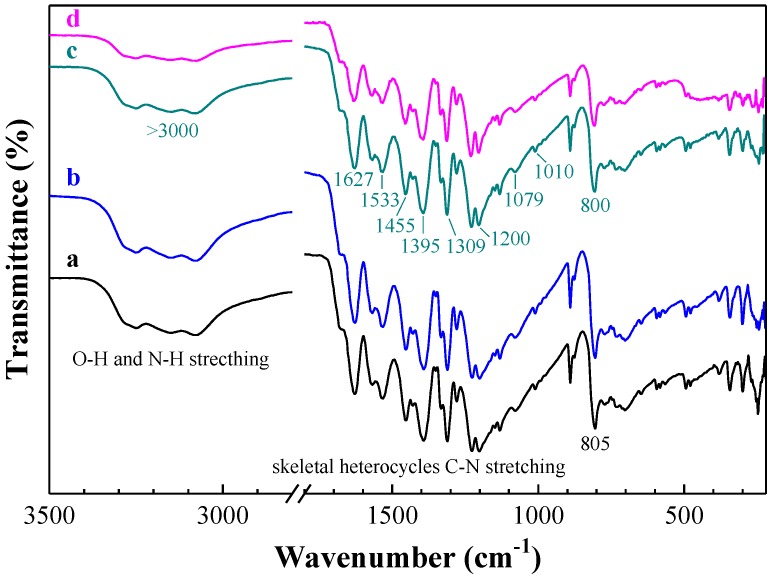
ATR-FTIR spectra of: (**a**) pristine g-C_3_N_4_, (**b**) protonated g-C_3_N_4_, (**c**) hydroxylaminated g-C_3_N_4_, and (**d**) hydrazinated g-C_3_N_4_.

The spectra of the protonated g-C_3_N_4_ samples revealed similar characteristic features to those found in the as-prepared g-C_3_N_4_, thus confirming that the structural integrity of g-C_3_N_4_ remained intact after protonation (Figure 2). Strong absorption bands could be observed in the 1200-1650 cm^−1^ range, arising from the skeletal stretching of C–N heterocycles and comprising both trigonal (N–(C)3) (full condensation) and bridging C–NH–C units (partial condensation) [37]. This would point at a successful development of an extended C–N–C network. The broad band ranging from 3000 to 3700 cm^−1^ was assigned to N–H and O–H stretching, due to the free amino groups and adsorbed hydroxyl species, respectively, whereas the sharp band at ca. 805 cm^−1^ was originated from the breathing vibration of tri-*s*-triazine units [38].

After oxygen plasma treatment, all the characteristic bands were retained (Figure 2), featuring bands at 800 cm^−1^ (assigned to the tri-*s*-triazine ring); and at 1200, 1309, 1395, 1455, 1533, and 1627 cm^−1^ (related to C–NH–C and N–(C) stretching modes). The peaks located at 1010 cm^−1^, 1079 cm^−1^, and above 3000 cm^−1^ corresponded to signals from N–OH groups. These results indicated that the oxygen plasma treatment led to the incorporation of N–OH on the surface of the C_3_N_4_.

The treatment of g-C_3_N_4_ with hydrazine monohydrate (Figure 2) appeared to decrease the signal intensity of all peaks with respect to the original untreated g-C_3_N_4_ sample or to that treated with oxygen plasma.

#### 3.1.2. ATR-FTIR Spectra of Matrimid/g-C_3_N_4_ MMMs

**Figure 3:**
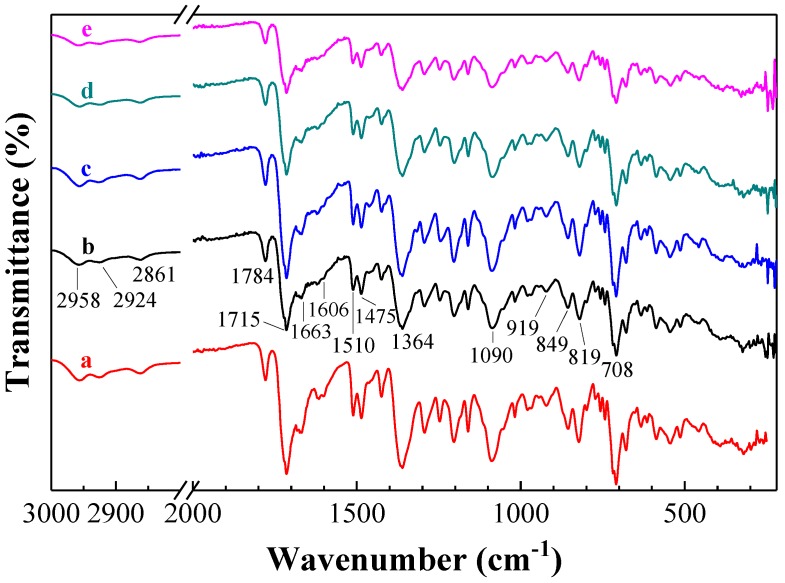
ATR-FTIR spectra of (**a**) a pristine Matrimid matrix and of Matrimid MMMs doped at 2 wt % with different fillers: (**b**) as-prepared g-C_3_N_4_, (**c**) protonated g-C_3_N_4_, (**d**) oxygen plasma-treated g-C_3_N_4_, and (**e**) hydrazinated g-C_3_N_4_.

A first comparison of the spectra of pure Matrimid and of the Matrimid MMMs doped with as-prepared or protonated g-C_3_N_4_ (Figure 3) evidenced shifts in the bands at 819 cm^−1^ (shifted to 824 cm^−1^), 849 cm^−1^ (to 858 cm^−1^), 919 cm^−1^ (to 924 cm^−1^), 1475 cm^−1^ (to 1489 cm^−1^), and 1606 cm^−1^ (to 1618 cm^−1^). These shifts were sufficient to suggest an interaction between Matrimid and g-C_3_N_4_. As regards other strong bands in the doped samples, they were found at 708 cm^−1^ (v(CNC) out-of-plane bending); 1090 cm^−1^ (v(CNC) transversal stretching); 1364 cm^−1^ (v(CNC) axial stretching); 1510 cm^−1^ (v(C=C) aromatic stretching); 1663 cm^−1^ (v(C=O) benzophenone carbonyl stretching); 1715 cm^−1^ and 1784 cm^−1^ (v(C=O) symmetric and asymmetric stretching, respectively); 2861 and 2924 cm^−1^ (aliphatic C–H stretching); and 2958 cm^−1^ (aromatic C–H stretching).

Differences in the nature of the dopant, i.e., protonated vs. non-protonated g-C_3_N_4_, mainly affected the band at 1672 cm^−1^ (amide C=O stretching), which, after protonation, was shifted to 1663 cm^−1^. This finding is important because it suggests that the carbonyl groups in Matrimid can act as electron acceptors from –NH^+^ units from exfoliated protonated g-C_3_N_4_, resulting in a material assembled through electrostatic interactions (this matter will be discussed in detail in Section 3.4).

In relation to the ATR-FTIR spectra of MMMs doped with oxygen plasma and hydrazine treated g-C_3_N_4_, also depicted in Figure 3, it is worth noting that no bands associated with the N–OH or –NHNH_2_ groups of g-C_3_N_4_ (which would result from those treatments) were observed. This may be due to low dopant doses, and would be in agreement with the findings of Chen et al. [35], who reported no remarkable differences between pristine g-C_3_N_4_ and g-C_3_N_4_-NHNH_2_. On the other hand, the band at 1606 cm^−1^, present in the pristine matrix, was found to disappear upon doping in both cases.

### 3.2. SEM Analysis

SEM was used to study the MMMs cross-section morphology. The micrographs of the neat Matrimid membrane (not shown) featured a smooth surface without obvious imperfections, in good agreement with those reported, for instance, by Ebadi Amooghin et al. [39].

**Figure 4:**
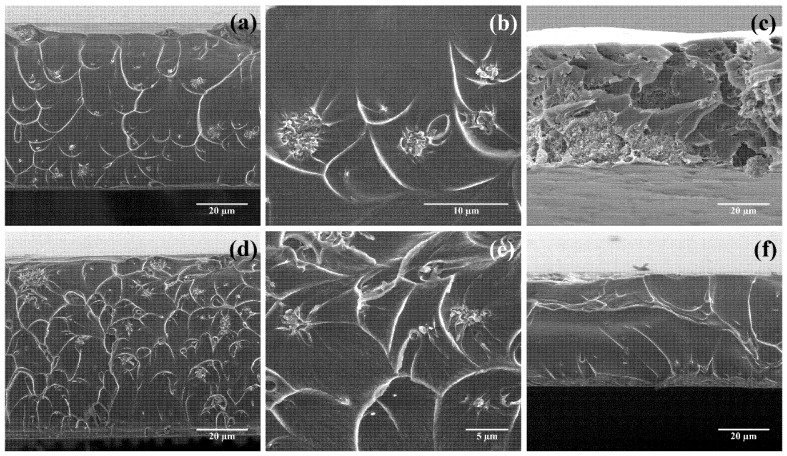
Cross-sectional SEM micrographs for MMMs doped with g-C_3_N_4_ subjected to different treatments and prepared with different sonication times: Matrimid membrane doped with 0.5 wt % protonated g-C_3_N_4_ at 3000× (**a**) and 6000× (**b**) magnification; Matrimid membrane doped with 10 wt % protonated g-C_3_N_4_ loading after 4 hours of sonication (**c**) at 3000×; Matrimid loaded with 0.5 wt % g-C_3_N_4_ treated with oxygen plasma at 3000× (**d**) and 10000× (**e**); Matrimid loaded with 0.5 wt % hydrazine monohydrate-treated g-C_3_N_4_ at 3000× (**f**). Micrographs of MMMs at 2 wt % loading were analogous to the ones at 0.5 wt %.

Representative micrographs of Matrimid/g-C_3_N_4_ MMMs with different g-C_3_N_4_ loadings and sonication times are shown in Figure 4a–c. At low loadings (0.5 wt %, Figure 4a,b) and sonication times (30 min), the g-C_3_N_4_ dopant was typically well dispersed. However, at higher loadings (e.g., 10 wt %, Figure 4c) and moderate sonication times (4 h), several large clusters of g-C_3_N_4_ could be found. These clusters ranged in size from 20 to 50 nm. This would be in agreement with the findings of Tian et al. [23], who evaluated the improvement of gas separation performance of mixed matrix membranes composed of PIM-1 doped with different g-C_3_N_4_ loads as compared to pure PIM-1 membranes. They found that, using CHCl_3_ as a solvent and after sonication for 12 h, the g-C_3_N_4_ dopant was well dispersed throughout the matrix at low loading levels (0.5–1 wt %), and also observed the presence of partial agglomeration for higher filler contents (>1 wt %).

It is also worth noting that, in contrast with the neat Matrimid membrane, a scalloped morphology was observed for the MMMs doped with protonated g-C_3_N_4_ (Figure 4a,b). As noted by Venna et al. [40] (and references therein), it may be attributed to the formation of elongated polymer segments with increased plastic deformation of the polymer and can be regarded as an indication of a good interaction between the polymer and the filler, further supported by the absence of a sieve-in-a-cage morphology (i.e., when there are interfacial voids larger than the penetrating molecules, so a by-pass around the particles is produced, enhancing the permeability and reducing the apparent selectivity [41]) at each loading.

SEM analysis was also conducted upon Matrimid doping with 0.5 wt % of g-C_3_N_4_ treated with oxygen plasma (Figure 4d,e) or with hydrazine monohydrate (Figure 4f). In the hydroxylaminated g-C_3_N_4_-doped samples, one could observe a crater-like pattern in which the eye of each crater was formed by nanosized fillers, similar to that obtained for protonated g-C_3_N_4_, albeit more evident. The filler also appeared to be uniformly distributed along the polymer matrix. According to Ebadi Amooghin et al. [39], such crater-like morphology of MMMs arises from an adequate compatibility existing between two phases, possibly due to the interfacial stress concentrations because of the polymer matrix/filler consecutive debonding. Thus, this deformation of the polymer matrix was again indicative of a strong interaction of polymer chains and filler. On the other hand, the MMMs doped with hydrazinolyzed g-C_3_N_4_ did not show a significant modification of the smooth aspect of pristine Matrimid, thus suggesting a weaker interaction.

### 3.3. Studies on Thermal Stability of the Matrimid/g-C_3_N_4_ Membranes

**Figure 5:**
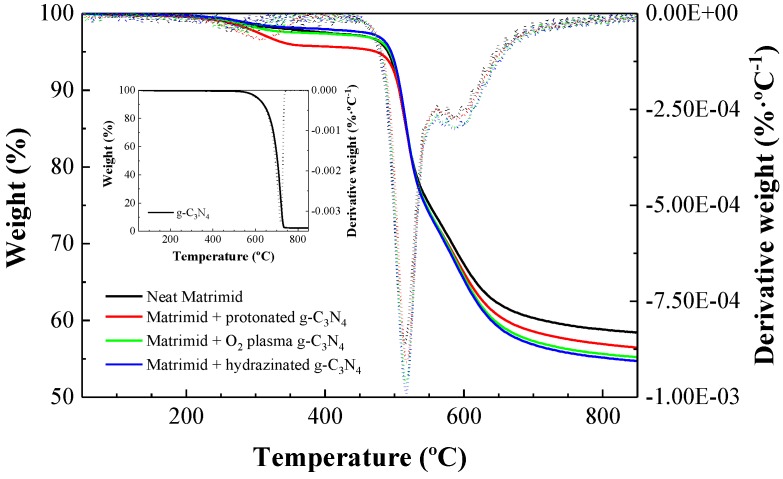
TG (solid line, *y*-axis on the left side of the graph) and DTG (dashed line, *y*-axis on the right side of the graph) curves for neat Matrimid and Matrimid-based membranes doped with 0.5 wt % of protonated g-C_3_N_4_, hydroxylaminated g-C_3_N_4_, and hydrazinated g-C_3_N_4_. The inset shows the TG/DTG curves for the untreated g-C_3_N_4_ filler.

The thermal stability of the Matrimid/g-C_3_N_4_ membranes was evaluated in comparison to pristine Matrimid (Figure 5). The thermograms showed that all the studied membranes were stable up to 470 °C and that only a small weight loss was observed between 200 °C and 250 °C (see Table 1), which could be attributed to residual solvent evaporation (the boiling point of DMAc is 202–204 °C). The inflection point for this first stage occurred at 351 °C for pristine Matrimid, and at lower temperatures for the MMMs membranes: 295 °C for Matrimid loaded with protonated g-C_3_N_4_, 247 °C for Matrimid doped with oxygen plasm-treated g-C_3_N_4_, and 287 °C for Matrimid with hydrazine-treated g-C_3_N_4_. Above 450 °C, a decomposition in two stages took place, with inflection points at around 517 °C and 591 °C (for pristine Matrimid they appeared at 518 °C and 580 °C, respectively). This second weight loss could be ascribed to the evolution of CO, CO_2_, and CH_4_ from the cleavage of the benzene ring pattern of the Matrimid matrix. Degradation of the Matrimid/g-C_3_N_4_ composites ended at 850 °C after a brief carbonization process.

Taking in account the delayed TG inflection points for MMMs vs. neat Matrimid at around 591 °C (588 °C for Matrimid/hydrazinated g-C_3_N_4_; 589 °C for Matrimid/hydroxylaminated g-C_3_N_4_ and 593 °C for Matrimid/protonated g-C_3_N_4_ vs. 580 °C for pristine Matrimid, as shown in Figure 5), it may be inferred that thermally stable structures were created in the membrane matrix after treatment with either g-C_3_N_4_(N–OH) or g-C_3_N_4_(N–H^+^).

### 3.4. CO_2_/CH_4_ and O_2_/N_2_ Gas Separation Performance of Matrimid/g-C_3_N_4_ MMMs

Apropos of the pristine Matrimid membrane, the CO_2_/CH_4_ selectivity value was within the range reported in other recent works (34.2 [42], 34.9 [43], 35.7 [44], 36.3 [45]), and so was the O_2_/N_2_ selectivity (6.5–6.8 [42,46,47]).

Doping of the Matrimid MMMs with protonated g-C_3_N particulates at 0.5 wt % increased the CO_2_/CH_4_ selectivity by 36.9% (from 36.3 to 49.6) as compared to the neat Matrimid membrane, although the O_2_/N_2_ selectivity decreased by 5.7% (from 7.0 to 6.6). Doping at 2 wt % led to a lower increase in the CO_2_/CH_4_ selectivity (by 16.5%, from 36.3 to 42.2), but the O_2_/N_2_ selectivity was preserved (Table 2).

The behavior for the MMMs doped with oxygen plasma-treated g-C_3_N_4_ treatment as a function of the filler doping values was the opposite: doping at 2 wt % led to a larger increase in the CO_2_/CH_4_ selectivity (by 52.2%, from 36.3 to 55.2) than doping at 0.5 wt % (4.4% increase). On the other hand, doping at 0.5 wt % led to a significant enhancement of O_2_/N_2_ selectivity (26.3% increase, from 7.0 to 8.9), while the increase for 2 wt % doping was only 2.7%.

In relation to the hydrazine treatment, it enhanced CO_2_/CH_4_ selectivities (by 7.5% and by 11.4% for 0.5 wt % and 2 wt % doping, respectively), while O_2_/N_2_ separation values remained similar to those of the Matrimid matrix (0.7% and −3.7% variation).

The results in the two latter treatments would be in agreement with observations by other authors, such as Tian et al. [23], who synthesized MMMs with a PIM matrix doped with g-C_3_N_4_, and an increase in the permeability values was observed as the filler concentration was increased to reach >1 wt %.

The enhancement of gas separation in the Matrimid membranes after doping with modified g-C_3_N_4_ may be referred either to an increase of hydrogen bonding in the Matrimid/g-C_3_N_4_ composite structure or to cross-linking modifications in Matrimid mediated by g-C_3_N_4_.

**Figure 6:**
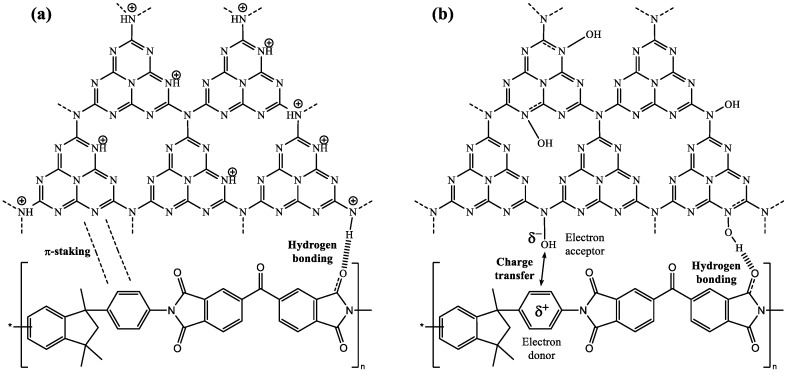
(**a**) Linking modes between protonated g-C_3_N_4_ and Matrimid; (**b**) a possible linking mode between oxygen plasma-treated g-C_3_N_4_ and Matrimid.

The first hypothesis suggests that the good results obtained for Matrimid MMMs doped with protonated g-C_3_N_4_ would be referred to, on the one hand, their exfoliated nature and the associated surface gain for interaction, and, on the other hand, to the presence of –NH^+^ groups capable of bonding, through hydrogen bonding, with the carbonyl groups of the Matrimid matrix. The benzene rings of both g-C_3_N_4_ and Matrimid would lend themselves to the formation of “π-staking” (Figure 6a).

In the case of g-C_3_N_4_ treated with oxygen plasma, designed to provide hydroxylamine N–OH groups that would bond with the carbonyl groups of Matrimid, it is unclear whether interactions between filler and matrix would be mediated by subsequent hydrogen bonding between such groups. There is also the possibility of formation of charge transfer between the –OH (behaving as electron acceptor group) and the aromatic nucleus in Matrimid (behaving as an electron donor group), as depicted in Figure 6b.

In the case of Matrimid/hydrazinated g-C_3_N_4_ MMM, its behavior can be associated to a potential cross-linking effect of the g-C_3_N_4_(NH–NH_2_) filler. Addition of H_2_N–R–NH_2_ fillers has been reported to lead to cross-linking modification of polyimides [49], increasing chain packing and inhibiting the intra-segmental and inter-segmental mobilities of the matrix, resulting in higher gas selectivity [50]. For instance, *p*-phenylenediamine (PPD) was reported to be a good cross-linking agent for Matrimid, showing a remarkable enhancement of selectivity for O_2_/N_2_ as compared to the unmodified membrane (from 6.2 to 10.0 due to the affinity of nitrogen-containing molecules towards oxygen) [13]. Thus, the hydrazinated g-C_3_N_4_ discussed herein could be regarded as an alternative to PPD, albeit the associated enhancement of gas selectivity for O_2_/N_2_ would be lower.

## 4. Conclusions

Modified g-C_3_N_4_ was assessed as a filler for the doping of Matrimid matrices with a view to enhancing their gas separation properties. The resulting Matrimid/g-C_3_N_4_ MMMs were characterized through ATR-FTIR vibrational analysis, SEM microscopy, thermal analysis techniques, and gas perm-selectivity assays. Due to the strong interfacial interactions among the modified g-C_3_N_4_ and the Matrimid matrix, the hybrid nanocomposite membranes featured high swelling resistance and mechanical stability. In the assays presented herein, the doping of Matrimid with modified g-C_3_N_4_ led to an enhancement of CO_2_/CH_4_ selectivity—as compared to that of the pure Matrimid membrane—in all cases. In particular, MMMs doped with 0.5 wt % protonated g-C_3_N_4_ and with 2 wt % hydroxylaminated g-C_3_N_4_ showed the best CO_2_/CH_4_ separation performances (with an increase by 36.9% and 52.2% vs. neat Matrimid, respectively). On the other hand, doping with 0.5 wt % oxygen plasma-treated g-C_3_N_4_ improved the O_2_/N_2_ selectivity by 26.3%, as compared to pristine Matrimid. Thus, the chemical modification of g-C_3_N_4_ may hold promise as an efficient pathway toward the doping of Matrimid and other membranes.

## Figures and Tables

**Table 1:** TG curves features for neat Matrimid, the g-C_3_N_4_ filler and the three MMMs under study

Pristine Matrimid		g-C_3_N_4_		Protonated g-C_3_N_4_		Oxygen Plasma-Treated g-C_3_N_4_		Hydrazinated g-C_3_N_4_	
Weight Loss (%)	Midpoint (°C)	Weight Loss (%)	Midpoint (°C)	Weight Loss (%)	Midpoint (°C)	Weight Loss (%)	Midpoint (°C)	Weight Loss (%)	Midpoint (°C)
2.8	287	97.6	700	4.2	29.4	2.70	284	2.2	280
23.4	516			23.3	518	24.8	518	25.9	517
14.9	609			15.9	611	17.0	611	17.1	611

**Table 2:** Permeabilities/selectivities of the Matrimid/g-C_3_N_4_ MMMs for He, N_2_, O_2_, CH_4_, CO_2_, O_2_/N_2_, and CO_2_/CH_4_ as a function of different filler treatments at different doping levels (0.5 wt % and 2 wt %)

MMM	Permeability (Barrer)					Selectivity		Ref.
	He	N_2_	O_2_	CH_4_	CO_2_	O_2_/N_2_	CO_2_/CH_4_	
Matrimid (control)	22.00	0.27	1.90	0.24	8.70	7.0	36.3	[48]
Matrimid/protonated g-C_3_N_4_ 0.5 wt %	23.53	0.26	1.70	0.15	7.69	6.6	49.6	This study
Matrimid/protonated g-C_3_N_4_ 2 wt %	20.17	0.20	1.44	0.15	6.45	7.1	42.2	This study
Matrimid/hydroxylaminated g-C_3_N_4_ 0.5 wt %	24.12	0.19	1.71	0.21	7.86	8.9	37.9	This study
Matrimid/hydroxylaminated g-C_3_N_4_ 2 wt %	23.63	0.23	1.69	0.13	7.15	7.2	55.2	This study
Matrimid/hydrazinated g-C_3_N_4_ 0.5 wt %	20.60	0.20	1.44	0.17	6.56	7.1	39.0	This study
Matrimid/hydrazinated g-C_3_N_4_ 2 wt %	18.65	0.20	1.36	0.15	6.19	6.8	40.4	This study
